# A Systematic Investigation of Polymer Influence on Core Scale Wettability Aided by Positron Emission Tomography Imaging

**DOI:** 10.3390/polym14225050

**Published:** 2022-11-21

**Authors:** Bergit Brattekås, Martine Folgerø Sandnes, Marianne Steinsbø, Jacquelin E. Cobos

**Affiliations:** 1 Department of Physics and Technology, University of Bergen, 5007 Bergen, Norway; 2 Department of Chemistry and Bioscience, Aalborg University, 6700 Esbjerg, Denmark

**Keywords:** HPAM, polymer, porous media, wettability, wetting stability

## Abstract

Polymers have been used as viscosifying agents in enhanced oil recovery applications for decades, but their influence on rock surface wettability is rarely discussed relative to its importance: wettability largely controls fluid flow in porous media and changes in wettability may significantly influence subsequent system performance. This paper presents a two-part systematic investigation of wettability alteration during polymer injection into oil-wet limestone. The first part of the paper determines wettability and wetting stability on the core scale. The well-established Amott–Harvey method is used, and five full cycles performed with repeated spontaneous imbibition and forced displacements. Wettability alterations are measured in a polymer/oil system, to determine polymer influence on wettability, and evaluated towards simpler brine/oil and glycerol/oil systems, to determine reproducibility and uncertainty related to the method and fluid/rock system. Polymer injection into oil-wet limestone core plugs is shown to repeatedly and reproducibly reverse the core wettability towards water-wet. Wettability changed both quicker and towards stronger water-wet conditions with polymer solution as the aqueous phase compared to brine and glycerol. The second part of the paper attempts to explain the observed behavior; by utilizing in situ imaging by Positron Emission Tomography, an emerging imaging technology within the geosciences. High resolution imaging provides insight into fluid flow dynamics during water and polymer injections, identifying uneven displacement fronts and significant polymer adsorption.

## 1. Introduction

Polymer flooding was presented as an oil recovery technique as early as the 1960s [1] and has since become a proven and widely used enhanced oil recovery (EOR) method. The main purpose of polymers in EOR is to increase the viscosity of the injected water phase [2], hence improving the mobility ratio between water and displaced oil. Polymer floods in porous media have been shown to improve volumetric sweep efficiency and the recovery of bypassed oil [3], and is also reported to decrease viscous fingering [4], enhance flow between vertical heterogeneous layers [4] and increase pull-out in dead end pores [5,6]; all of which are connected to the rheological properties of the polymer solution. 

Polymer floods are also known to influence porous media flow properties due to interactions between the polymer solution and the rock surface. Interactions play an important role in polymer flooding efficiency and may cause changes in both the pore space (clogged pores and decreased pore sizes) and rock surface characteristics. The effects of polymer/rock interactions include disproportionate permeability reduction (DPR); where a polymer flood subsequently reduces water relative permeability without having a similar effect on oil relative permeability (see [7,8]), polymer retention (see [9]) and wettability changes. The interaction effects are often different sides of the same story, hence, the wettability parameter has been discussed alongside DPR and retention. Because wettability largely controls fluid flow in porous rock, including saturation functions and end points, and wettability changes strongly influence system performance, wettability should also be treated as a single important parameter. Wettability-focused experimental studies have shown that polymers may alter rock surface wettability, but few core plugs are often used and the results are missing comparison to a water/oil baseline. Barreau et al. [10] injected brine, mineral oil, and high molecular weight, non-ionic polyacrylamide (PAM) into water-wet and oil-wet (Silane-treated) sandstone core plugs. Capillary pressure, relative permeability and end-point saturations were measured before and after polymer injection and observed to change. At water-wet conditions, changes were mainly attributed to polymer adsorption and associated pore size reductions, and the presence of adsorbed polymer was observed to increase the capillary pressure across the mobile saturation range. At initially oil-wet conditions the capillary pressure values changed from negative to positive: hence indicating a full reversal of wettability from oil-wet to water-wet. DPR was observed, with a greater reduction in water relative permeability compared to oil and was more pronounced in the water-wet medium. Askarinezhad et al. [11], on the other hand, investigated polymer-based DPR treatments in one water-wet and one oil-wet sandstone core and found that the DPR effect was stronger in the oil-wet compared to the water-wet core. Elmkies et al. [12] injected a non-ionic polyacrylamide polymer into slightly water-wet and slightly oil-wet St-Maximin and Estaillades limestone and found that adsorption of the hydrophilic polymer within the core plugs partly restored the cores to the initial water-wet state. Surface adsorption of thick polymer layers was visually verified by Grattoni et al. [13] in glass micro-models. Hatzignatiou et al. [14] assessed the influence of wettability on polymer flooding efficiency in one water-wet and one oil-wet (Quilon-treated) Berea sandstone core. Changes in wettability were qualitatively assessed based on breakthrough times and normalized recovery. They found that polymer retention was low in oil-wet porous media and attributed this effect to wettability, where polymer/rock interactions were limited by the oil film covering the surface and adsorption minimized. Hatzignatiou et al. [9] added Bentheimer core material to the study. The wettability change was determined by interpreting the oil production and pressure drop across the core during waterfloods, and showed that the wettability alteration of the Bentheimer core may not have been successful. The study, however, noted that retention was notably higher in the water-wet Berea compared to the Bentheimer core: which shows that the permeability, surface area and pore size distribution in the core material is also of importance when investigating polymer/rock interactions on the core scale. Juarez-Morejon et al. [15] used spontaneous imbibition experiments to evaluate wettability alteration by hydrolyzed polyacrylamide (HPAM) polymer in intermediate-wet Bentheimer sandstone. Amott tests in brine and polymer solutions were performed and showed that polymer produced oil more efficiently by spontaneous imbibition than brine. Oil recovery was also improved when polymer imbibition was performed after spontaneous brine imbibition. End-point wetting indices for brine (I_AH_ = 0.08) and polymer (I_AH_ = 0.81) systems, indicated stronger water-wet conditions with polymer present in the pore network. 

This paper quantifies changes in wettability caused by the polymer injection in several subsequent floods, and seeks to systematically evaluate the wetting influence of HPAM polymer on the wetting preference and stability of oil-wet limestone. Previous studies have often used sandstone cores; only a few cores have been used in each study, and the wetting stability of the system has not been evaluated. The Edwards limestone core material was chosen for this study due to previous reproducibility in spontaneous imbibition experiments [16] and a fairly stable wetting condition after ageing [17]. In the first part of the paper we use a simple laboratory approach and repeated Amott–Harvey cycles to assess wettability and wetting stability. Three aqueous fluids: HPAM polymer solution, glycerol solution and synthetic brine, were paired with the same mineral oil and results compared. The core scale study confirms that hydrophilic HPAM polymer alters the wetting preference of oil-wet core plugs towards water-wet conditions much quicker than brine or glycerol solutions; an effect that is reproducible across several core plugs. In the second part of the paper, in situ visualization by Positron Emission Tomography (PET) imaging was used during cyclic injection of radioactively labelled water or polymer and oil. PET imaging provides insight into dynamic fluid displacements and influences on the sub-core scale [18] and revealed the existence of a low-flow region in the core, promoting heterogeneous displacements. Polymer injection improved the displacement pattern, as expected, but severely constricted the flow of both oil and water after placement. Imaging confirm that a significant amount of polymer remains adsorbed or otherwise entrapped in the pore space after displacement by oil and water. 

## 2. Materials and Methods

### 2.1. Core Preparations

**Figure 1:**
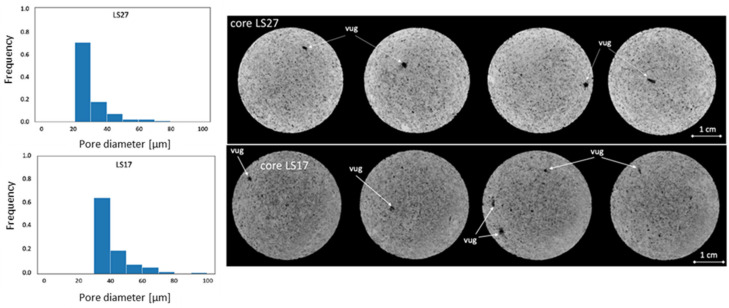
Pore size distribution (**left**) and micro-CT images (**right**) of two Edwards limestone cores: one of lower (LS27) and one of higher (LS17) permeability. The spatial resolution of images was 25 µm, and smaller pores are, hence, not visible in the images. The majority of pores were below 50 µm in diameter.

Nine cylindrical core plugs of Edwards limestone core material (Edwards plateau in West Texas) were prepared. The cores were gently washed with tap water and dried for one week at 60 °C. The cores were saturated with synthetic brine (mixing 40 g NaCl, 34 g CaCl_2_, 5 g MgCl_2_ in 1000 g of distilled water and sodium azide (NaN_3_) (0.05 mL) was added to prevent bacterial growth [19]) under vacuum, and porosity was measured gravimetrically. Absolute permeabilities were found by Darcy’s law, after measuring the differential pressure across cores during water flooding at several volumetric flow rates. Measured porosity and permeability values were in the range of 21–28% and 18–52 mD, respectively (Table 1), which agreed with previously measured values [16]. A ProCon X-ray CT-ALPHA computed tomography (CT) scanner was used to gain insight into the rock structure of two cores in separate ends of the permeability range: LS17 (K = 52.6 mD) and LS27 (K = 18.5 mD) (Figure 1), and confirmed the heterogeneous nature of Edwards limestone. A variety of pore sizes were visible, including vugs. More vugs were present in core LS17, and the maximum vug diameter was higher in at 315 µm compared to 270 µm for core LS27, which could contribute to a higher measured permeability. 

### 2.2. Wettability Alteration

Oil-wet limestone was used in all experiments; initially water-wet outcrop core plugs were aged dynamically to oil-wet conditions, using an aging procedure previously shown to yield uniform wettability distributions [20,21]. Crude oil from a North Sea carbonate reservoir was used (details of the oil properties and composition given in [17]). Prior to aging the crude oil was flooded through a limestone filter to remove impurities and oxidized for 3 days at 80 °C. Fully water-saturated core plugs were placed in a Hassler biaxial core holder inside a heating cabinet at 80 °C and drained to uniform irreducible water saturation (S_iw_) by injecting 2.5 pore volumes (PV) of crude oil both directions at a differential pressure of 2 bar/cm core length. Crude oil was thereafter continuously injected at a low flow rate. The flow direction was reversed mid-way in the aging process. An aging time of 144 h (6 days) was used for all core plugs, and was reproducibly shown to render weakly oil-wet conditions [17] for the used crude oil/brine/rock system. The crude oil was miscibly exchanged by decalin (decahydronaphtalene), and subsequently by mineral oil n-Decane to end the aging process. N-decane constituted the oil phase during further studies at ambient conditions. 

### 2.3. Imbibing Fluids

Core wetting conditions after aging and wettability alterations during cyclic floods were measured during five consecutive Amott–Harvey cycles, using three different fluid couples: (1) synthetic brine/mineral oil, (2) glycerol solution/mineral oil, and (3) HPAM polymer solution/mineral oil. Synthetic brine/mineral oil was used as a baseline in this work, as most reported spontaneous imbibition tests at ambient temperature are performed with this or similar combinations. Amott–Harvey cycles to estimate wettability alterations were performed at ambient temperature, where n-Decane viscosity and density were 0.92 cP and 0.73 g/cm^3^, respectively, and brine viscosity and density were 1.09 cP and 1.05 g/cm^3^, respectively. 

**Figure 2:**
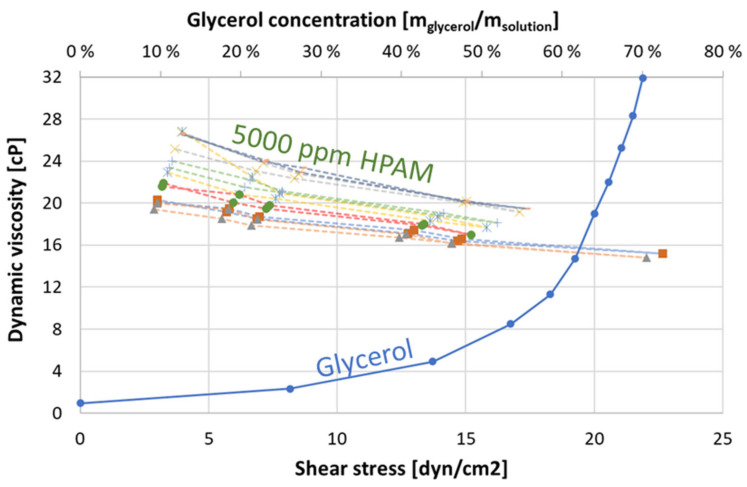
Viscosity (*y*-axis) as a function of shear stress for seven different 5000 ppm polymer solutions (primary *x*-axis), and as a function of concentration for glycerol solutions (secondary *x*-axis).

Glycerol solutions were used as high-viscous aqueous fluid phases to resemble the polymer/mineral oil mobility ratio, but do not contain polar components that could adsorb to mineral surfaces to influence wettability. Glycerol solution/mineral oil therefore constitutes a second baseline, to evaluate viscosity effects in the presented procedure. Glycerol solutions were made by mixing brine with a predetermined volume of pure glycerol on a magnetic stirrer, and filtering was not necessary. Glycerol solutions exhibit Newtonian behavior, and viscosity was solely a function of Glycerol concentration (Figure 2): the concentration of glycerol ranged between 61–69 wt% to produce viscosities resembling the polymer solution (viscosity range 13.6–24.9 cP).

Partially hydrolyzed polyacrylamide (HPAM) (5 million Daltons molecular weight) mixed in brine at 5000 ppm concentration constituted the polymer solution in all experiments. Microgels and other nonlinear, multimolecular structures were removed by filtering (filter pore size 5 μm) [22]. Filtered polymer solutions were clear and transparent, with a density similar to brine, due to the high water content. Seven batches of polymer solutions at 5000 ppm concentration were made, and dynamic viscosities were measured using a Brookfield DV-II+Pro Viscometer (Model LVDV-II+Pro) at ambient, constant temperature conditions. Solution viscosity was measured at several spindle speeds (increasing, then decreasing). Polymer solutions used for imbibition were measured several times. The viscosity of twenty production effluent samples was also measured to evaluate whether fluid properties changed when passing through porous media. The viscosity as a function of shear stress for the different polymer solutions (Figure 2) shows non-Newtonian shear-thinning behavior as expected. Viscosity hysteresis was observed at low shear stress for five different solutions. Viscosities measured at the highest shear stress were used for imbibition scaling and end-point relative permeability calculations.

### 2.4. PART 1: Core Scale Evaluation of Wetting Stability

Changes in spontaneous imbibition characteristics were used to evaluate wettability alterations in limestone core plugs. Scaling equations were necessary to compare spontaneous imbibition with varying aqueous viscosity, and to correct small core dimension variations [23]. The scaling equation developed by Ma et al. [24] accounts for differences in viscosity and was used in this work (see Appendix A). 

Because the core plugs used were not strongly wetted, saturation end points were not reached during spontaneous imbibition, and forced injections were performed to obtain end point saturations. The wettability indices of water and oil are quantified by measuring the increase in water and oil saturation during spontaneous imbibition ($∆S_{ws}$ and $∆S_{os}$) and the overall increase in water and oil saturation ($\Delta S_{wt}$ and $\Delta S_{ot}$) after forced displacement. A full Amott–Harvey cycle consisted of spontaneous imbibition and forced injection of aqueous phase to measure the water index ($I_{w}$),

$$I_{w}=\frac{∆S_{ws}}{\Delta S_{wt}}\;$$

and spontaneous imbibition and forced injection of oil to measure the oil index ($I_{o}$),

$$I_{o}=\frac{∆S_{os}}{\Delta S_{ot}}$$

**Figure A1:**
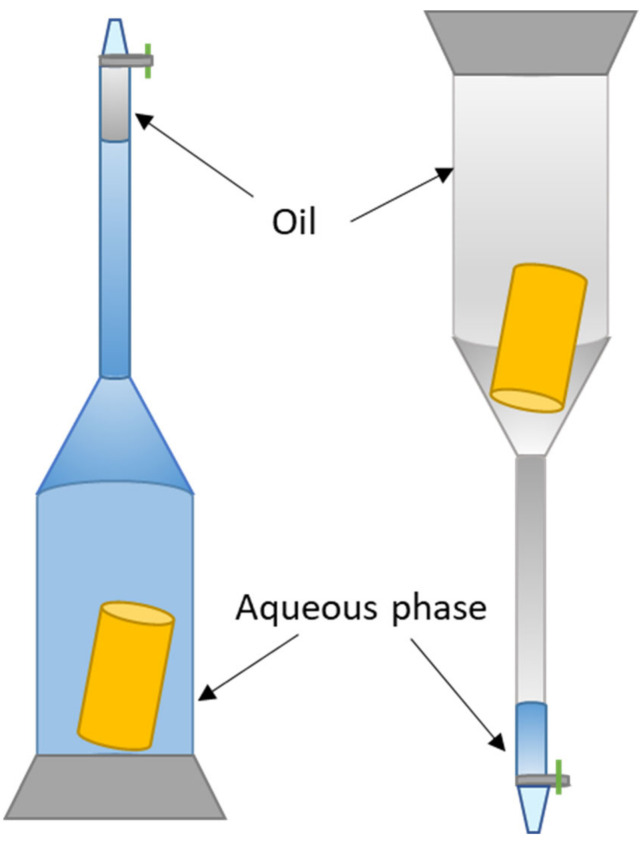
Amott cells used for spontaneous imbibition of water (**left**) and oil (**right**). Spontaneous imbibition was performed at ambient conditions.

The water- and oil indices were used to calculate the Amott–Harvey index $I_{AH}$ = $I_{w}$ − $I_{o}$. Five consecutive Amott–Harvey cycles were performed to evaluate wettability stability and wettability alteration caused by polymer compared to glycerol and brine. An overview of the fluids used in each cycle is presented in Table 2. Each cycle consisted of the following experimental steps:

*Spontaneous aqueous phase imbibition:* Aged core plugs at *S_iw_* were placed in glass imbibition cells filled with an aqueous fluid (brine, glycerol, or polymer solution). All faces open (AFO) boundary conditions were used, i.e., the entire core plug surface was open to spontaneous imbibition. The volume of produced oil was measured as a function of time. The imbibition cells were gently rolled before each volume measurement to release oil drops from the core surface or glass wall, and the accumulated oil volume was recorded. The cores remained in the imbibition cells for minimum 168 h, or until the spontaneous imbibition process had ended and the produced volume was stable for several measuring points (days). The experimental setup may be found in the Appendix A (Figure A1).

**Figure A2:**
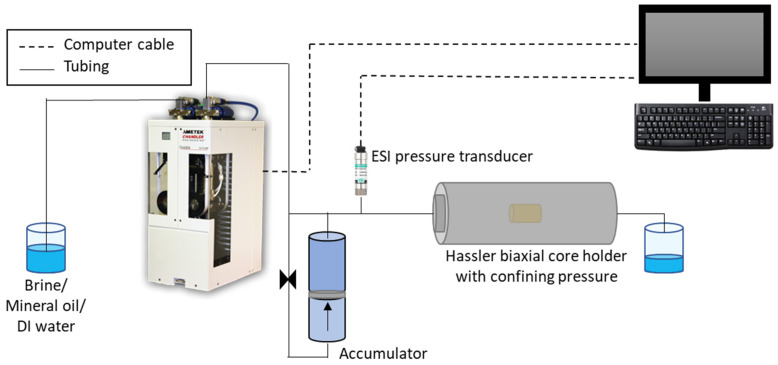
Schematic of the experimental setup used for forced imbibition and drainage. Forced fluid injections were performed at ambient conditions.

*Forced aqueous phase imbibition*: The core plugs were placed in a Hassler biaxial core holder and the aqueous phase was injected at constant pressure (2 bar/cm), often corresponding to high injection rates. Effluent production was measured as a function of time, and the saturation development monitored by material balance. Injection was performed through both end faces of the core alternately, to maintain a uniform saturation. Brine was used in the pump directly (Quizix QX), while an accumulator containing either polymer or glycerol was connected between the pump and core holder during forced injections of higher-viscosity fluids. Polymer/glycerol solutions yielded much lower flow rates (5 to 20 mL/h) than brine injection (ranging upwards from 100 mL/h). At least 1.5 PV of polymer/glycerol solution was injected in each direction. When the production of oil stopped and the core was at residual oil saturation (*S_or_*), the end-point relative permeability for water was measured: the aqueous phase was injected at three consecutive constant rates while measuring the differential pressure. The end-point relative permeability was calculated through a common generalization of Darcy’s law (the ratio of the effective permeability of a fluid to the absolute permeability of the rock. A longer period of time was needed to obtain stable differential pressures for each rate when high-viscous polymer or glycerol solution was injected (hours, compared to minutes for brine). The experimental setup may be found in the Appendix A (Figure A2).

*Spontaneous oleic phase imbibition:* Core plugs at *S_or_* were placed in imbibition cells filled with mineral oil. The imbibition cells were rotated with the graded cylinder pointing downwards, to facilitate volume recordings of produced aqueous phase (heavier than imbibing oil). The volume of produced aqueous phase was measured as a function of time, and the imbibition cells were gently rolled before each volume measurement. The cores remained in the imbibition cells for minimum 168 h, or until the spontaneous imbibition process had ended and the produced volume was stable for several measuring points (days). 

*Forced oleic phase imbibition*: The core plugs were placed in a Hassler biaxial core holder and the oleic phase was injected at constant pressure (2 bar/cm), often corresponding to high flow rates (ranging between 100–900 mL/h). Aqueous phase recovery was recorded as a function of time and saturation monitored by material balance. Injection was performed through both end faces of the core alternately, to maintain a uniform end point saturation along the core length. When the production of aqueous phase had stopped and the core was at the irreducible water saturation (*S_iw_*), the end-point relative permeability for oil was measured by constant rate injection at three different rates.

### 2.5. PART 2: In Situ Visualization of Viscous Displacements in Oil-Wet Limestone

Two full Amott–Harvey cycles were performed on limestone core LS27 to assess initial wettability. The core plug was thereafter placed in a specially made core holder to visualize viscous displacements using PET imaging. PET imaging detects radioactive decay, hence radioactive tracers were used to label the injected fluid phases. In this paper we used Fluorodeoxyglucose (^18^F-FDG) to label brine and polymer solution before injection. ^18^F-FDG is miscible in water and approximately 0.5ml was used to label 150 mL of aqueous solution before injection: hence the ^18^F-FDG is not likely to influence the density and viscosity of the injected fluid. The labelled aqueous phase is explicitly traced through the porous medium, while the oleic phase is implicitly imaged during the subsequent. The core was placed in the PET scanner at S_wi_ and consecutive fluid injections were performed (Table 3): (1) a baseline brine injection followed by oil, (2) polymer solution injection followed by oil, and (3) a second brine injection followed by oil. For further details about the PET imaging setup, we refer to [18].

## 3. Results

### 3.1. PART 1: Core Scale Evaluation of Wetting Stability

The relative permeability end points and wetting indices for five consecutive Amott–Harvey cycles are presented in Table 4. The following sections of this paper describes the dynamic spontaneous imbibition processes and indicated wettability changes in further detail. 

#### 3.1.1. First Cycle (Brine/Oil)

**Figure 3:**
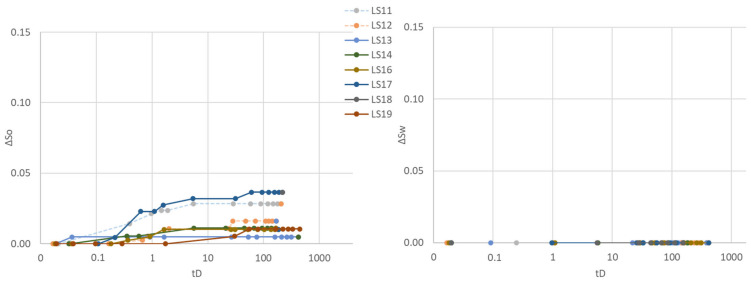
Spontaneous saturation development as functions of dimensionless time during imbibition. Cores were placed in imbibition cells with oil and brine, respectively, during the first Amott–Harvey cycle. (**Left**): Oil saturation (∆S_o_) during spontaneous oil imbibition, and (**Right**): Water saturation (∆S_w_) during spontaneous water imbibition. Note that the *y*-axis only represents a part of the mobile saturation range. The scale is kept constant for all cycles, to enable simple comparison of results.

Brine/mineral oil was used for all cores during the first Amott–Harvey cycle, to determine the wettability achieved during 144 h of dynamic aging. Mineral oil spontaneously imbibed into all core plugs (Figure 3), while water indices were zero; i.e., the cores did not spontaneously imbibe brine. Negative Amott–Harvey indices of −0.01 to −0.08 were measured, confirming near neutral wet to weakly oil-wet conditions. 

#### 3.1.2. Second Cycle

**Figure 4:**
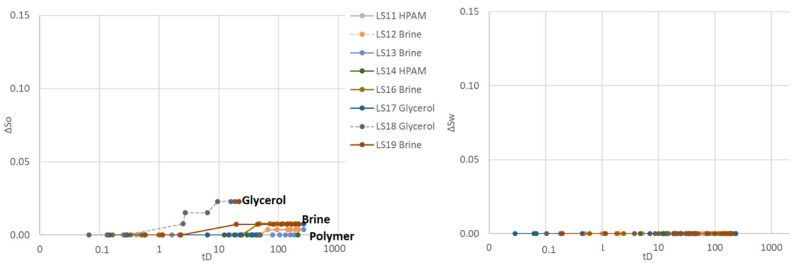
Spontaneous saturation development as functions of dimensionless time during the second Amott–Harvey cycle. Cores were placed in imbibition cells with oil and brine, glycerol, or polymer solution. (**Left**): Oil saturation (∆S_o_) during spontaneous oil imbibition, and (**Right**): Water saturation (∆S_w_) during spontaneous aqueous phase imbibition. Note that the *y*-axis only represents a part of the mobile saturation range. The scale is kept constant for all cycles, to enable simple comparison of results.

Three different aqueous fluids were used in the second cycle: polymer (LS11 and LS14), glycerol (LS17 and LS18) and brine (LS12, LS13, LS16 and LS19), all paired with the same mineral oil (n-Decane). Spontaneous aqueous phase imbibition was not recorded in any of the core plugs (Figure 4). Spontaneous oil imbibition was observed in core plugs exposed to and saturated by brine and glycerol, and the Amott–Harvey index varied between I_AH2_ = −0.01 and −0.05. One of the glycerol saturated core plugs (LS17) did not imbibe oil, nor glycerol and I_AH2_ = 0. Imbibition of oil was not recorded in polymer saturated core plugs LS11 and LS14, obtaining an Amott–Harvey index of zero, I_AH2_ = 0. After spontaneous oil imbibition, the core plugs were brought back to irreducible water saturation by forced oil displacement. The Amott–Harvey method is insensitive around neutral-wet conditions (small volumes imbibed), and it is not possible to defer from the two first cycles whether the change in imbibed volume is an effect of wettability, mobility, or other factors.

#### 3.1.3. Third Cycle

**Figure 5:**
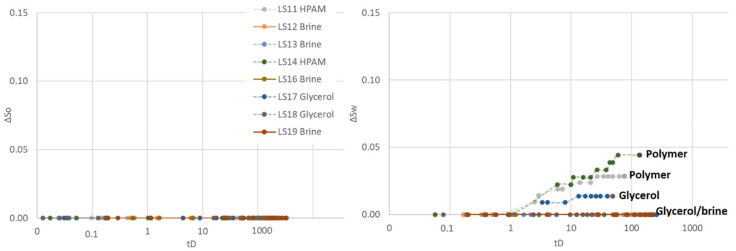
Spontaneous saturation development as functions of dimensionless time during the third Amott–Harvey cycle. Cores were placed in imbibition cells with oil and brine, glycerol, or polymer solution. (**Left**): Oil saturation (∆S_o_) during spontaneous oil imbibition, and (**Right**): Water saturation (∆S_w_) during spontaneous aqueous phase imbibition. Note that the *y*-axis only represents a part of the mobile saturation range. The scale is kept constant for all cycles, to enable simple comparison of results.

Spontaneous aqueous phase imbibition was observed for the first time in the third Amott–Harvey cycle, prevailing in the polymer saturated cores LS11 and LS14. Some imbibition of glycerol was recorded in LS17, while no imbibition of aqueous phase was visually observed or recorded in the four brine saturated cores or glycerol saturated LS18 (Figure 5). Before oil imbibition, brine was injected to displace high-viscous polymer in LS14 and glycerol solution in LS18. Glycerol is expected to be diluted and miscibly displaced from LS18, while polymer may remain as an adsorbed layer along the pore walls in LS14. The objective was to assess whether polymer adsorption caused the observed wettability changes, or if the results were significantly influenced by the high polymer viscosity. Spontaneous oil imbibition was not recorded in any of the core plugs during the third cycle, hence Amott–Harvey indices were I_AH3_ = 0 for brine saturated cores, I_AH3_ = 0 to 0.04 for glycerol saturated cores and convincingly positive at I_AH3_ = 0.08–0.19 for polymer saturated cores. 

#### 3.1.4. Fourth Cycle

**Figure 6:**
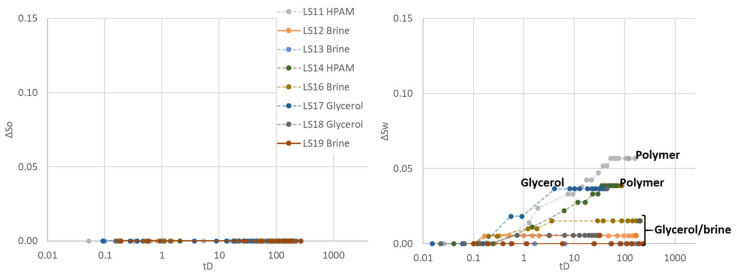
Oil (**left**) and water (**right**) saturation change as a function of dimensionless time during the fourth Amott–Harvey cycle. Note that the *y*-axis only represents a part of the mobile saturation range. The scale is kept constant for all cycles, to enable simple comparison of results.

Spontaneous oil imbibition was not recorded for any cores during the fourth Amott–Harvey cycle. Spontaneous aqueous phase imbibition was, however, recorded in most cores (Figure 6). Cores containing polymer exhibited weakly water-wet conditions during this cycle: polymer saturated core LS11 had the highest spontaneous saturation change and the highest positive I_AH_ 0f 0.27. Core LS14 had a slightly lower I_AH_ of 0.16: polymer was displaced by brine during the third cycle for this core, and it should be noted that the measured I_AH_ changed significantly in core LS11 (continuously exposed to polymer) but did not change in LS14 (core exposed to and flooded by polymer, which is displaced by brine before oil imbibition). Similarly, more glycerol imbibed into glycerol saturated core LS17 than into LS18 (glycerol displaced by brine before oil imbibition). Some of the baseline brine/oil saturated cores also started imbibing small volumes of the aqueous phase, hence achieving zero (LS13 and LS19) or slightly positive I_AH_ (0.01 for LS12 and 0.03 for LS16). 

#### 3.1.5. Fifth Cycle

**Figure 7:**
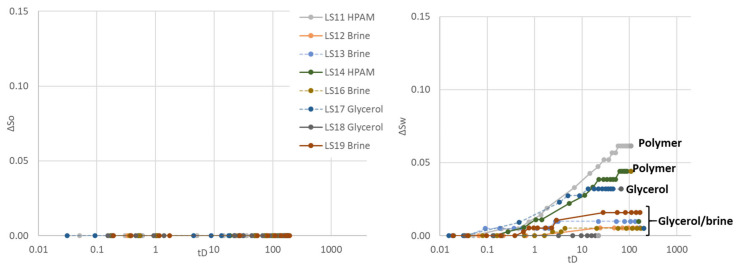
Oil (**left**) and water (**right**) saturation change as a function of dimensionless time during the fifth Amott–Harvey cycle. Note that LS11 and LS14 were placed in Amott cells filled with polymer or glycerol for spontaneous imbibition, and were also forcedly flooded with polymer/glycerol. After flooding, however, brine was injected to displace the high viscous aqueous phase and oil imbibition were performed in water-filled cores. Note that the *y*-axis only represents a part of the mobile saturation range. The scale is kept constant for all cycles, to enable simple comparison of results.

Aqueous phase spontaneous imbibition also occurred during cycle five, and was comparable to the previous cycle (Figure 7). Spontaneous oil imbibition was not recorded in any of the cores. Polymer Amott–Harvey indices were I_AH_ = 0.27 (LS11) and I_AH_ = 0.18 (LS14), remarkably comparable to cycle four and indicative of weakly water-wet conditions. Cores containing glycerol also had comparable indices to cycle 4, I_AH_ = 0.09 (LS17) and I_AH_ = 0 (LS18), which corresponds to near neutral wet or weakly water-wet conditions. Baseline brine/oil cores exhibited wetting indices of 0.01–0.03, i.e., near neutral wetting conditions.

#### 3.1.6. Estimating Wettability Alterations from Spontaneous Imbibition

**Figure 8:**
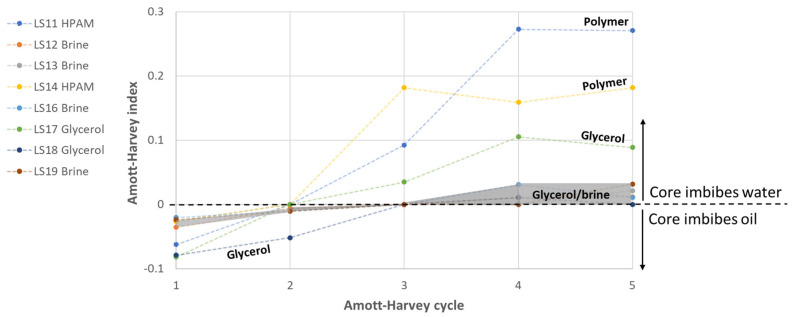
Wettability development during five Amott–Harvey cycles where the aqueous phase was polymer, glycerol, or brine (shaded area). The brine/oil cores were previously described in [17], and the figure modified from [26].

Measured Amott–Harvey indices for all five cycles are shown in Figure 8, where the grey shaded area represents baseline cores (synthetic brine/mineral oil). Baseline wettability slowly changed from weakly oil-wet to weakly water-wet conditions, (further described in [17]). Note that quantification of wettability by the Amott–Harvey wettability index is highly uncertain at near neutral wetting conditions due to small volumes imbibed, and the inherent uncertainty in wetting indices is therefore high. A qualitative change in wettability was, however, apparent by this simple observation: all cores spontaneously imbibed oil during the first Amott–Harvey cycle, but not brine. With an increasing number of Amott–Harvey cycles, the cores first stopped spontaneously imbibing oil, and later started spontaneously imbibing brine. We can therefore conclude that the wetting index changed from negative to positive in the course of five Amott–Harvey cycles, although the wetting indices cannot be unambiguously determined. 

The presence of polymer in the pore space enhanced the wettability alteration: oil spontaneous imbibition immediately ceased when the cores were in contact with polymer, and polymer spontaneous imbibition started in the next cycle. The volume of aqueous phase spontaneously imbibed was much higher when the cores were placed in polymer-filled Amott cells, compared to brine-filled cells. The rapid increase in volume indicates that the presence of polymer in the pore space contributed to wettability being reversed from slightly oil-wet towards weakly water-wet conditions. Similar observations were previously made by other authors [12,15,25] but without comparing the results to a brine/oil baseline. Our thorough study shows oil spontaneous imbibition in all brine saturated cores during the first two Amott–Harvey cycles. During the fourth and fifth (of five consecutive) Amott–Harvey cycles, some cores (not all) started spontaneously imbibing brine. In cores where polymer was introduced, oil spontaneous imbibition immediately stopped, and aqueous phase imbibition was observed during the following cycle; already imbibing more than four times the end-point volume of brine. Our spontaneous imbibition study shows that wettability changed both quicker and towards stronger water-wet conditions with polymer solution as the aqueous phase. 

#### 3.1.7. Observations of End-Point Relative Permeability 

**Figure 9:**
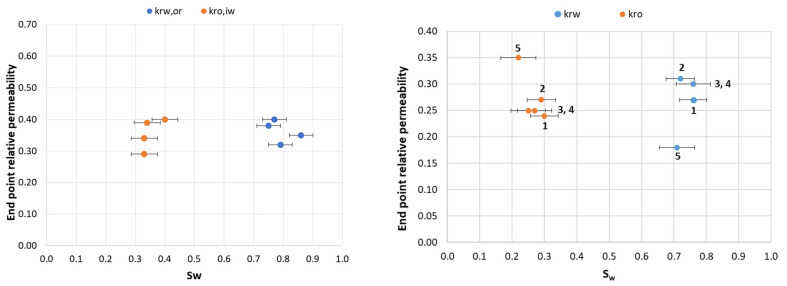
End-point relative permeabilities to water and oil as a function of water saturation for (**left**): LS11, LS14, LS17, and LS18 during the first Amott–Harvey cycle. (**right):** development in end point relative permeabilities in core LS19 during five consecutive Amott–Harvey cycles. Note the *y*-axis scale.

End-point relative permeability can be another measure for wettability and wettability alterations at the core scale [27,28]. Polymer injection is, however, regularly observed to decrease the relative permeability to water more than that to oil; which must be understood in the interpretation of experiments. Initial end-point relative permeability measurements (oil/brine systems) supported slightly oil-wet to neutral wet conditions; with higher values for water than oil in 5 of 8 core plugs (Table 4). Most values ranged between 0.3 and 0.4, further supporting nearly neutral or slightly wetting conditions. Figure 9 plots end point relative permeabilities towards saturation for brine/oil baselines. The general behavior in brine/oil systems during the five consecutive Amott–Harvey cycles was well represented by core LS19: end point relative permeabilities were relatively stable in cycles 1–4, while k_ro_ increased and k_rw_ decreased in the fifth cycle, indicating a shift towards more water-wetting conditions. Spontaneous imbibition properties showed a slow and slight change towards water-wet conditions during the fourth and fifth cycles.

**Figure 10:**
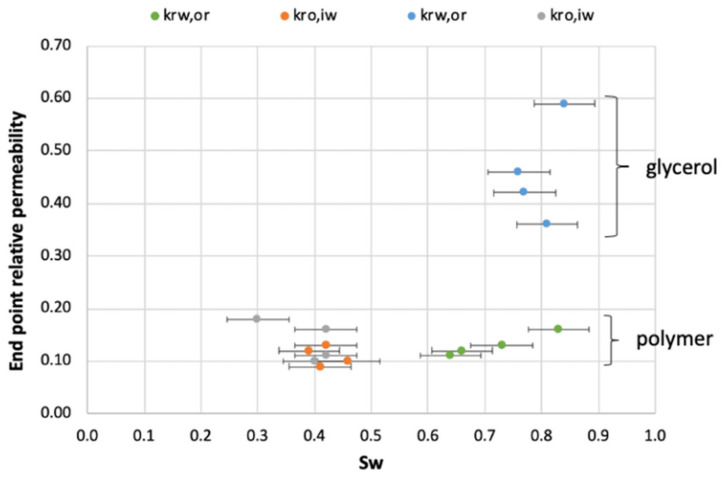
End-point relative permeabilities as a function of aqueous phase saturation (S_w_).

Introducing glycerol and polymer solutions as aqueous phases significantly influenced end point relative permeabilities, exemplified in Figure 10. Polymer solutions strongly inhibited the movement of both oil and water through the cores. Glycerol also inhibited the movement of oil, but improved the relative permeability for water. The validity of relative permeability measurements as wetting indicators in this case should not serve as support for solid conclusions; however, two experiments may provide insight into the role of polymer adsorption in our measurements, and are important to the overall findings. Cores LS14 and LS18 were flooded with brine after high-viscous aqueous phase injection, but are otherwise comparable to cores LS11 and LS17 where polymer and glycerol remained in the pore network. Aqueous phase end-points in core LS11 ranged from 0.11–0.16 after polymer injection, and remained fairly stable through four cycles. When polymer was displaced by water (core LS14) k_rw,or_ significantly decreased to below 0.02, indicating a significant resistance to water flow in the core. In the glycerol/oil system, aqueous phase end-points reverted to the initial level during water injection. The very low relative permeability end points encountered after polymer displacement in core LS14 indicate polymer adsorption within the core; water was not able to displace polymer residing near the pore walls, which may influence surface wetting conditions and/or decrease pore sizes (i.e., increase the capillary pressure). Measured oil relative permeability end-points were low after introduction of polymer into the two cores (0.09–0.13), and did not change when water was injected to displace polymer; hence, changes in pore structure/wettability inferred during polymer injection did not appear to be revertible in these experiments. 

### 3.2. PART 2: In Situ Visualization of Viscous Displacements in Oil-Wet Limestone 

In situ PET imaging was used to visualize cyclic brine, oil and polymer displacements on the core and sub-core level; to support whole core analysis by material balance (Part 1). Several cycles of aqueous and oleic fluid injection were performed in an oil-wet limestone core, where the aqueous fluid was radioactively labelled and explicitly and dynamically traced by PET imaging. Core L27 was mounted into a biaxial core holder for imaging by PET [18]. The injection schedule is shown in Table 3 along with initial and end point fluid saturations for each cycle. The PET signal was acquired during injections and was post-processed to produce three-dimensional images at predetermined time steps. A temporal resolution of three minutes was chosen for this study, and found to accurately capture displacement dynamics at the given flow rates. This section describes observations from PET imaging during cyclic injection into the core. Core L27 was prior to imaging confirmed to be oil-wet through two full Amott–Harvey cycles (I_AH,1_ = −0.21, I_AH,2_ = −0.06). 

#### 3.2.1. First Water and Oil Flood

**Figure 11:**
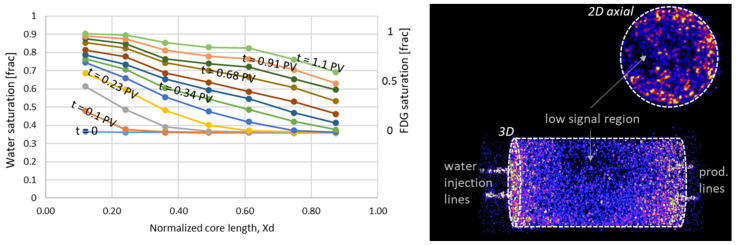
(**Left**) 1D saturation profiles for core LS27 during the first waterflood, showing a less than ideal displacement. Oil recovery was efficient until water breakthrough (t = 0.39 PV), and continued to increase until 1.1 PV of water was injected. No change in saturation was observed between t = 1.1–2 PV injected. (**Right**) A 3D rendering of the flooded core at static, no-flow conditions show a low-signal region in the core middle, which was not efficiently flooded by water.

PET imaging revealed an uneven water displacement front during initial waterflooding, rapidly moving through the core. Water breakthrough was observed at t = 0.39 PV injected, confirmed by effluent measurements. In situ imaging showed that the water saturation close to the inlet end face was high, with a steadily decreasing trend towards the outlet (Figure 11). The water saturation increased from 0.36 to 0.79 during the flood according to effluent measurements and the end point relative permeability to water was 0.21; hence, few hints of an unfavorable displacement is visible in global data. By PET imaging, a low signal region could, however, be observed close to one side of the core circumference, at dimensionless core length *X_d_* = 0.3–0.6, indicating a less efficient displacement in this part of the core (Figure 11). 

**Figure 12:**
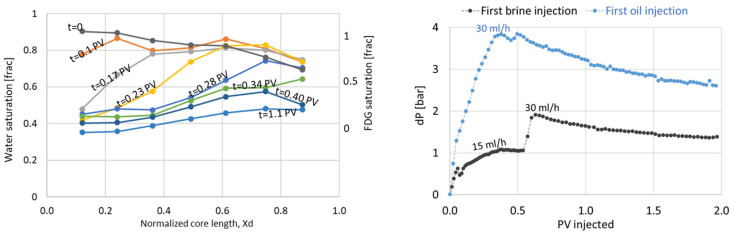
(**Left**) Saturation profiles during oil displacement in core L27. (**Right**) Pressure profiles during the first water and oil floods in core L27.

Oil was injected to displace radioactive water and was only implicitly quantifiable by PET imaging. The water saturation decreased from *S_w_ =* 0.79 to 0.38 during oil flooding according to effluent measurements. The radioactive signal decreased throughout the core; in all locations where a radioactive signal was initially present. The oil displacement front appeared closer to piston-like than water displacement, and only minor variations in saturation were detectable by PET after oil breakthrough at the outlet (t = 0.34 PV). Achieved differential pressures (Figure 12) were much lower during waterflooding compared to oil flooding- indicating that water flows freely through the largest pores, as expected at oil-wet conditions.

#### 3.2.2. Polymer and Subsequent oil Flood

**Figure 13:**
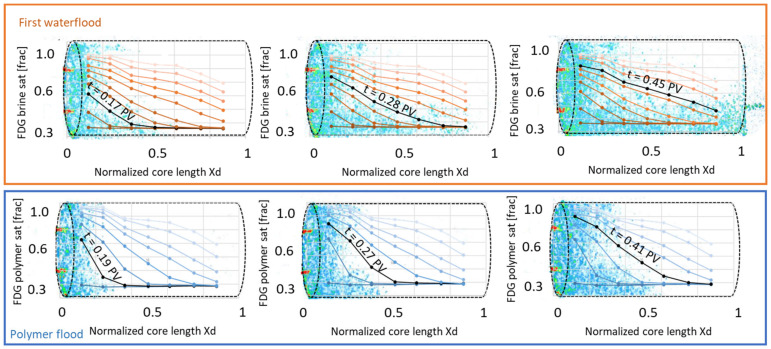
Comparison of sequential water and polymer injection in the same oil-wet limestone core plug.

**Figure 14:**
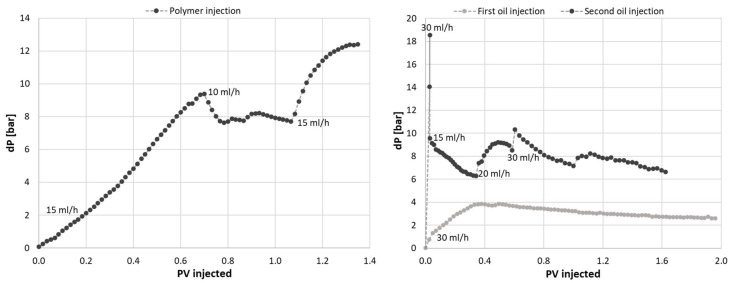
Differential pressure across the core plug during polymer injection (**left**) and subsequent oil injection (**right**), compared to the initial oil flood.

Polymer displacement through the core was, as expected, more even, with a slower break-through and a higher end-point saturation (Figure 13). The PET signal (equivalent to polymer saturation during this flood) in the low-flow region close to the core circumference was higher during polymer flooding compared to the waterflood, but remained low relative to the end point saturation; i.e., the zone was still less efficiently flooded than the remaining core. The differential pressure increased almost linearly during polymer flooding (Figure 14) due to the high viscosity of the polymer solution, and the injection rate was reduced to 10 mL/h after t = 0.66 PV to avoid harm to laboratory equipment with inherent pressure limitations. The injection pressure levelled off and fluctuated around 8 bar until t = 1.08 PV injected. When the injection rate was increased back to the original level, the differential pressure increased up to a maximum level of 12.4 bar, and remained stable at this value until injection ended (t = 1.37 PV).

**Figure 15:**
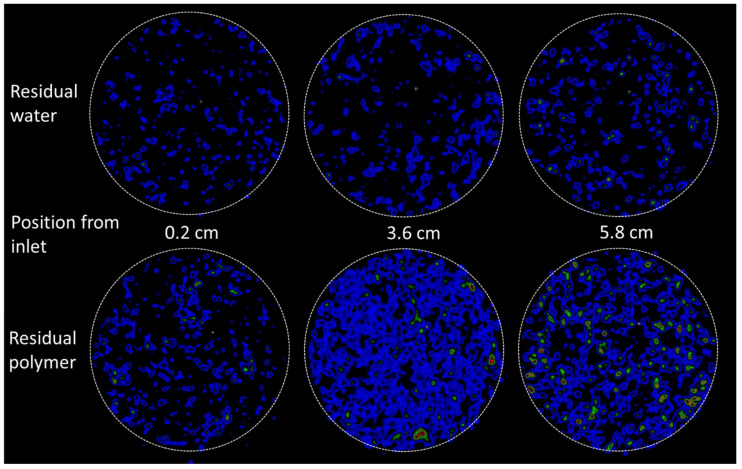
PET images of the core cross section comparing residual water (**top**) to residual polymer (**bottom**) in the core after long-term oil flooding. The images are corrected for radioactive decay and produced using the same threshold settings. The signal to noise ratio was 32.8 (water) and 34.4 (polymer) at this stage of injection. The rendered PET signal in the images should not be understood as quantitative saturation, but rather serve as a basis for comparison of residual fluids in oil flooded cores containing radioactive water versus radioactive polymer. Blue color indicates the presence of radioactivity (i.e., labelled aqueous phase), warmer colors indicate a higher concentration of aqueous fluid.

Oil was injected directly after the polymer flood, using the same injection rate as during the initial oil flood. The differential pressure (Figure 14) immediately increased to 18.6 bars; almost five times higher than during initial oil flooding. The oil injection rate was lowered to 15 mL/h to keep experimental equipment within pressure limitations, and was stepwise increased as the displacement pressure stabilized. After 1.6 PV of oil injected, the pressure was fairly stabilized at 6.6 bar using an injection rate of 30 mL/h. High pressures are expected during the displacement of high viscous polymer by low viscous oil, but it should be noted that the flow of oil also remained severely restricted after polymer displacement from the core: with a resulting end point relative permeability for oil measured at 0.04. PET imaging provided insight into the location of radioactively labelled polymer during oil flooding: while effluent measurements clearly illustrated a significant saturation change within the core from *S_w_* = 0.82–0.36, the PET signal interestingly remained high throughout the core. A small PET signal change did occur close to the inlet end face initially (coinciding with the very high initial oil injection pressure), but no clear displacement front was seen during continued oil flooding (Figure 15). Hence: radioactively labelled polymer remained in the pore network also after oil flooding. This supports the hypothesis of polymer adsorption and entrapment; which may change the apparent wettability from oil-wet (oil flow along pore walls, water flow in pore middle) to hydrophilic (polymer layer along walls, oil and water flow in pore middle). Additional insight into the location of fluids in relation to pore walls is not available by this imaging method. 

#### 3.2.3. Water and Oil Flood after Polymer

**Figure 16:**
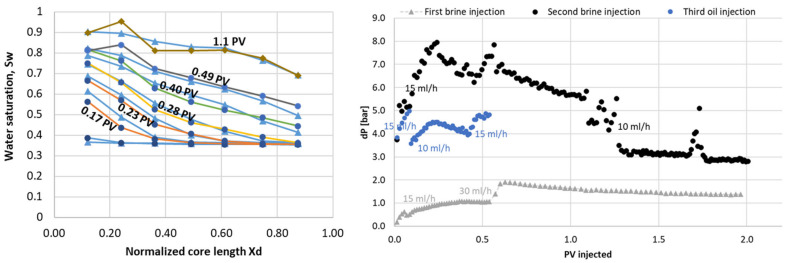
Water displacement after polymer was visualized by PET imaging. Saturation development may be seen in the left figure, while the differential pressure development is given on the right.

A second cycle of water and oil was injected to measure system parameters after the pore network had been exposed to polymer. Displacement patterns in the core were visible by PET during water and polymer injections, and showed that the two water displacements (before and after polymer) were very similar in terms of saturation development (Figure 16), with most flow occurring outside of the low-flow region. The measured differential pressure (Figure 16) was much higher and more unstable during the second waterflood compared to initial flooding. The elevated pressures cannot be explained by an unfavorable mobility ratio, although traces of polymer were visibly discharged in the production tubing directly following pressure peaks (pressure rising with subsequent immediate decline). The frequency of pressure disturbances declined after more than one pore volume of water had been injected, and remained fairly stable after t = 1.8 PV injected. The end point relative permeability to water was very low, at 0.04 compared to 0.21 during initial injection. The oil *k_ro,iw_* also remained low, at 0.03. This was not unique in core LS27, but a reproducible observation in all cores used in this study: low oil and water relative permeabilities were observed in all cores flooded with polymer solution (Table 4). 

## 4. Discussion

In situ imaging complemented the core scale experiments presented in Part 1 of this paper, and the measured saturation ranges and relative permeability end points corresponded well. We observed lower displacement efficiency in some regions of the core by PET, which may have several possible explanations, including: stronger wetting affiliation or smaller pores (both inducing higher entry capillary pressure), lower porosity (fewer pores), or clogging of pores by polymer, leading to fluid diversion. Micro-CT imaging of the core prior to ageing (Figure 1) did not indicate a non-uniform pore size distribution within the core, hence smaller or fewer pores are less likely explanations in this experiment. Because an uneven displacement was also observed during initial waterflooding, before polymer injection, polymer adsorption and clogging is also a less likely explanation. Irregular flow patterns are known to occur at low viscous flow rates, and may be exaggerated due to small scale heterogeneities; hence, the limestone core material with its inherent heterogeneity is prone to unfavorable displacement. Similar low-flow regions, as observed in LS27, may thus also be present in other cores; heavily influencing the true boundary conditions during spontaneous imbibition, and likely impacting the measurements. This observation calls for caution in similar studies when using global parameters such as oil production, pressure drops and breakthrough times to assess polymer behavior in porous media. The strong and reproducible effect of polymer on wettability in this and previous studies, however, also allude to strong interactions and significant effects that are not suppressed by artifacts in any of the investigated system. Hence, formation wettability should be considered when planning polymer injections for EOR, due to the significant influence of polymer on both the porous rock and its flow properties. 

## 5. Conclusions

- Amott–Harvey cycles were used to qualitatively determine wetting change in polymer/mineral oil systems over time. Although the method has its weaknesses (especially around near-neutral wetting) the shift from oil-wet to water-wet conditions could be marked when the core plugs stopped spontaneously imbibing oil and started spontaneously imbibing water.
- When polymer was introduced to the cores oil spontaneous imbibition immediately stopped, and aqueous phase imbibition was observed during the following cycle; already imbibing more than four times the end-point volume of brine.
- After introduction of polymer to the core plugs, end point relative permeabilities for oil and water decreased, and the cores stopped spontaneously imbibing oil.
- This spontaneous imbibition study shows that wettability changed both quicker and towards stronger water-wet conditions with polymer solutions as the aqueous phase.
- PET imaging showed a continuously high presence of polymer in the core, also after oil flooding.
- The observations from our experimental work support polymer adsorption and entrapment as important mechanisms for apparent wettability change during and after polymer treatments.

## Figures and Tables

**Table 1:** Basic core plug properties.

Core ID	Length ±0.01 [cm]	Diameter ±0.01 [cm]	Pore Volume ±0.01 [mL]	Porosity ±0.06 [%]	Permeability ±0.05 [mD]
LS11	6.80	3.80	21.16	27.44	45.88
LS12	6.90	3.80	18.67	23.85	26.23
LS13	6.90	3.80	20.42	26.09	35.06
LS14	6.91	3.80	18.10	23.09	23.59
LS16	6.71	3.80	19.72	25.92	33.74
LS17	6.81	3.80	21.91	28.37	52.64
LS18	6.69	3.80	17.41	22.95	21.49
LS19	6.79	3.80	18.99	24.66	35.97
LS27	6.91	3.80	17.65	22.52	18.48

**Table 2:** Overview of fluid pairs used during each Amott–Harvey cycle. Note that the cores were also used in pairs of similar properties, and duplicate experiments were performed to evaluate reproducibility. Exemplified, core pairs L11/L17 (high K = 46–53 mD) and L14/L18 (low K = 22–24 mD) were compared after HPAM polymer (LS11 and LS14) and glycerol (LS17 and LS18) cycles. High viscous polymer and glycerol was displaced by brine before spontaneous imbibition in the third and consecutive cycles in core LS14 and LS18 to assess imbibition and wettability dependency on mobility.

Core	Cycle_1_	Cycle_2_	Cycle_3_	Cycle_4_	Cycle_5_
LS11	Brine/oil	Polymer/oil	Polymer/oil	Polymer/oil	Polymer/oil
LS12	Brine/oil	Brine/oil	Brine/oil	Brine/oil	Brine/oil
LS13	Brine/oil	Brine/oil	Brine/oil	Brine/oil	Brine/oil
LS14	Brine/oil	Polymer/oil	Polymer+ brine/oil	Polymer+ brine/oil	Polymer+ brine/oil
LS16	Brine/oil	Brine/oil	Brine/oil	Brine/oil	Brine/oil
LS17	Brine/oil	Glycerol/oil	Glycerol/oil	Glycerol/oil	Glycerol/oil
LS18	Brine/oil	Glycerol/oil	Glycerol+ brine/oil	Glycerol+ brine/oil	Glycerol+ brine/oil
LS19	Brine/oil	Brine/oil	Brine/oil	Brine/oil	Brine/oil

**Table 3:** Experimental schedule during PET imaging. Note that PET imaging only detects radioactive signal and cannot give information about initial saturation distribution or fluid affinity (wetting). The mobile saturation interval remained relatively stable between injections.

Inj. Fluid	PET Visualization	Inj. Rate [mL/h]	Water Saturation from [frac] to	
Brine	Signal from ^18^F-FDG labelled brine	15, 30	0.36	0.79
Oil	Implicit, displacing labelled brine	30	0.79	0.38
Polymer	Signal from ^18^F-FDG labelled polymer solution	15, 10	0.38	0.82
Oil	Implicit, displacing labelled polymer	30, 15, 20, 30	0.82	0.36
Brine	Signal from ^18^F-FDG labelled brine	15, 10	0.36	0.71
Oil	Implicit, displacing labelled brine	15, 10, 15	0.71	0.36

**Table 4:** Core scale parameters measured during five consecutive Amott–Harvey cycles. Cores LS12, 13, 16, and 19 were previously detailed in [17]. * indicates the end point measurements after polymer/glycerol was displaced by water.

	Core	LS11	LS12	LS13	LS14	LS16	LS17	LS18	LS19
Cycle 1	Aqueous phase	Brine	Brine	Brine	Brine	Brine	Brine	Brine	Brine
	k_rw,or_	0.35	0.27	0.29	0.38	0.30	0.32	0.4	0.4
	k_ro,iw_	0.4	0.24	0.25	0.34	0.30	0.39	0.29	0.29
	I_w_	0	0	0	0	0	0	0	0
	I_o_	0.06	0.04	0.01	0.03	0.02	0.08	0.08	0.02
	I_AH_	−0.06	−0.04	−0.01	−0.03	−0.02	−0.08	−0.08	−0.02
Cycle 2	Aqueous phase	Polymer	Brine	Brine	Polymer	Brine	Glycerol	Glycerol	Brine
	k_rw,or_	0.16	0.31	0.27	0.23	0.31	0.59	0.61	0.27
	k_ro,iw_	0.13	0.27	0.32	0.12	0.34	0.16	0.12	0.38
	I_w_	0	0	0	0	0	0	0	0
	I_o_	0	0.01	0	0	0.01	0	0.05	0.01
	I_AH_	0	−0.01	0	0	−0.01	0	−0.05	−0.01
Cycle 3	Aqueous phase	Polymer	Brine	Brine	Polymer/brine	Brine	Glycerol	Glycerol/brine	Brine
	k_rw,or_	0.13	0.30	0.27	0.13	0.30	0.36	0.30	0.28
					0.01 *			0.29 *	
	k_ro,iw_	0.1	0.25	0.33	0.1	0.32	0.11	0.22	0.39
	I_w_	0.09	0	0	0.18	0	0.04	0	0
	I_o_	0	0	0	0	0	0	0	0
	I_AH_	0.09	0	0	0.18	0	0.04	0	0
Cycle 4	Aqueous phase	Polymer	Brine	Brine	Polymer/brine	Brine	Glycerol	Glycerol/brine	Brine
	k_rw,or_	0.12	0.30	0.27	0.11	0.31	0.42	0.45	0.28
					0.02 *			0.35 *	
	k_ro,iw_	0.09	0.26	0.28	0.11	0.31	0.1	0.27	0.38
	I_w_	0.27	0.01	0	0.16	0.03	0.11	0.01	0
	I_o_	0	0	0	0	0	0	0	0
	I_AH_	0.27	0.01	0	0.16	0.03	0.11	0.01	0
Cycle 5	Aqueous phase	Polymer	Brine	Brine	Polymer/brine	Brine	Glycerol	Glycerol/brine	Brine
	k_rw,or_	0.11	0.18	0.24	0.06	0.27	0.46	0.31	0.24
					0.02 *			0.28 *	
	k_ro,iw_	0.12	0.32	0.37	0.12	0.41	0.18	0.37	0.37
	I_w_	0.27	0.01	0.02	0.18	0.01	0.09	0	0.03
	I_o_	0	0	0	0	0	0	0	0
	I_AH_	0.27	0.01	0.02	0.18	0.01	0.09	0	0.03

## Data Availability

Background data sets will be made available by request.

## Appendix A

According to Ma et al. [24] dimensionless time (t_D_) is given by

$$t_{D}=t\sqrt{\frac{K}{\varphi }}\frac{\sigma }{\mu_{\mathrm{gm}}}\frac{1}{L_{C}^{2}}$$

where t is time, K is the absolute permeability, σ is the interfacial tension between the wetting and the non-wetting phase, φ is the porosity, μ_gm_ is the geometric mean of the water (μ_w_) and oil (μ_o_) viscosities

$$\mu_{\mathrm{gm}}=\sqrt{\mu_{w}\mu_{o}}$$

and L^2^_c_ is the characteristic length for AFO boundary conditions found by

$$L_{c}=\frac{L_{S}d_{S}}{2\sqrt{d_{S}^{2}+2L_{S}^{2}}}$$

where L_S_ and d_S_ is the length and diameter of the core plug, respectively.
